# Facilitated interprofessional implementation of a physical rehabilitation guideline for stroke in inpatient settings: process evaluation of a cluster randomized trial

**DOI:** 10.1186/s13012-017-0631-7

**Published:** 2017-08-01

**Authors:** Nancy M. Salbach, Sharon Wood-Dauphinee, Johanne Desrosiers, Janice J. Eng, Ian D. Graham, Susan B. Jaglal, Nicol Korner-Bitensky, Marilyn MacKay-Lyons, Nancy E. Mayo, Carol L. Richards, Robert W. Teasell, Merrick Zwarenstein, Mark T. Bayley, on behalf of the Stroke Canada Optimization of Rehabilitation By Evidence – Implementation Trial (SCORE-IT) Team

**Affiliations:** 1 Department of Physical Therapy, University of Toronto, 160-500 University Ave, Toronto, ON M5G 1V7 Canada; 2 School of Physical and Occupational Therapy, McGill University, 3630 Promenade Sir William Osler, Montreal, QC H3G 1Y5 Canada; 3 Faculty of Medicine and Health Sciences, Université de Sherbrooke, 3001, 12e avenue nord, Bureau FM-2208, Sherbrooke, QC J1H 5N4 Canada; 4 University of British Columbia, 212-2177 Wesbrook Mall, Vancouver, BC V6T 1Z3 Canada; 5 School of Epidemiology and Public Health, University of Ottawa, 600 Peter Morand Cres, Ottawa, K1G 5Z3 Canada; 6 School of Physiotherapy, Dalhousie University, Office 405 Forrest Building, 5869 University Avenue, PO Box 15000, Halifax, NS B3H 4R2 Canada; 7 Division of Clinical Epidemiology, Division of Geriatrics, McGill University Health Center, Royal Victoria Hospital Site, Ross Pavilion R4.29, 687 Pine Ave W, Montreal, QC H3A 1A1 Canada; 8 Department of Rehabilitation, Faculty of Medicine, Université Laval and Centre de recherche en réadaptation et intégration sociale (CIRRIS), Institut de réadaptation en déficience physique de Québec (IRDPQ) Site Hamel, 525 Boul. Wilfrid-Hamel Est, Québec, QC G1M 2S8 Canada; 9 Parkwood Institute, 550 Wellington Road, London, ON N6C 0A7 Canada; 10 Schulich School of Medicine and Dentistry, Western University, Western Centre for Public Health and Family Medicine, 1151 Richmond St, London, ON N6A 3K7 Canada; 11 Toronto Rehabilitation Institute-University Health Network, 550 University Avenue, room 3-131 (3-East) 3rd Floor University Wing, Toronto, ON M5G 2A2 Canada

**Keywords:** Implementation, Facilitation, Interprofessional, Stroke, Rehabilitation, Guideline, Randomized controlled trial, Cluster randomization, Knowledge translation, Process evaluation

## Abstract

**Background:**

The Stroke Canada Optimization of Rehabilitation by Evidence-Implementation Trial (SCORE-IT) showed that a facilitated knowledge translation (KT) approach to implementing a stroke rehabilitation guideline was more likely than passive strategies to improve functional walking capacity, but not gross manual dexterity, among patients in rehabilitation hospitals. This paper presents the results of a planned process evaluation designed to assess whether the type and number of recommended treatments implemented by stroke teams in each group would help to explain the results related to patient outcomes.

**Methods:**

As part of a cluster randomized trial, 20 rehabilitation units were stratified by language and allocated to a facilitated or passive KT intervention group. Sites in the facilitated group received the guideline with treatment protocols and funding for a part-time nurse and therapist facilitator who attended a 2-day training workshop and promoted guideline implementation for 16 months. Sites in the passive group received the guideline excluding treatment protocols. As part of a process evaluation, nurses, and occupational and physical therapists, blinded to study hypotheses, were asked to record their implementation of 18 recommended treatments targeting motor function, postural control and mobility using individualized patient checklists after treatment sessions for 2 weeks pre- and post-intervention. The percentage of patients receiving each treatment pre- and post-intervention and between groups was compared after adjusting for clustering and covariates in a random-effects logistic regression analysis.

**Results:**

Data on treatment implementation from nine and eight sites in the facilitated and passive KT group, respectively, were available for analysis. The facilitated KT intervention was associated with improved implementation of sit-to-stand (*p* = 0.028) and walking (*p* = 0.043) training while the passive KT intervention was associated with improved implementation of standing balance training (*p* = 0.037), after adjusting for clustering at patient and provider levels and covariates.

**Conclusions:**

Despite multiple strategies and resources, the facilitated KT intervention was unsuccessful in improving integration of 18 treatments concurrently. The facilitated approach may not have adequately addressed barriers to integrating numerous treatments simultaneously and complex treatments that were unfamiliar to providers.

**Trial registration:**

Unique identifier-NCT00359593

**Electronic supplementary material:**

The online version of this article (doi:10.1186/s13012-017-0631-7) contains supplementary material, which is available to authorized users.

## Background

Stroke is a chronic disabling condition [1] that is expected to affect an increasing number of individuals due to population growth and aging [2]. Clinical practice guidelines for inpatient stroke rehabilitation settings provide clear treatment recommendations aimed at improving motor function, postural control, and mobility [3–5]. Despite evidence that guideline adherence is associated with functional recovery [6] and patient satisfaction [7], studies conducted in Canada and in the UK reveal that guideline application is inconsistent [8–11].

To narrow these knowledge to practice gaps, the Knowledge to Action (KTA) Process [12] recommends an evaluation of barriers to knowledge use and tailoring of knowledge translation (KT) strategies to address those barriers. Research conducted by our team and others has shown that implementation of a stroke rehabilitation guideline in the inpatient hospital setting presents unique challenges [13, 14]. Owing to a broad research base [15, 16], stroke rehabilitation guidelines consist of an extensive number of treatment recommendations [3]. Although multiple efficacious treatments may be appropriate for a patient, there may be insufficient time to apply all of them thus requiring individual health professionals to prioritize and select [13]. This process is complicated by the recommendation to deliver stroke rehabilitation by a core interdisciplinary team consisting of physical and occupational therapists, nurses, and speech-language pathologists, physiatrists/physicians, social workers, and dietitians [3]. Team functioning and communication may affect how well members prioritize and coordinate implementation of treatment [13]. The complexity of efficacious treatments in terms of the number of steps and technical skill required also varies widely which has implications for education and training [13]. Finally, stroke can lead to heterogeneous profiles of sensorimotor, communication, and cognitive impairment that cause variable levels of disability. The type and magnitude of stroke-related deficits, the incidence of shoulder pain which occurs in almost 30% of patients [17], and patient preferences can further influence health providers’ decisions to apply a recommended treatment [13, 18].

A multi-component intervention is required to overcome the challenges to integrating stroke rehabilitation guidelines targeting improvement in motor function, postural control, and mobility. Facilitation, defined as “the process of enabling (making easier) the implementation of evidence into practice” (p. 579) [19, 20] is a recognized strategy and core component of the PARiHS (Promoting Action on Research Implementation in Health Services) framework [20–22] that could potentially enable collaboration within stroke teams to implement a comprehensive stroke rehabilitation guideline. Reviews of facilitation [19, 23] have characterized it as both an individual role incorporating project management and leadership and a process involving individuals and teams. Facilitators may use a range of strategies to assist individuals and teams to apply evidence in practice as facilitation should be tailored to the needs of the local context [19, 22, 23]. For example, a local facilitator may organize training sessions to address a need to build clinicians’ capacity to implement a specific treatment and check-in at regular intervals to help maintain motivation levels. Facilitators may also approach managers to enable the purchase of equipment or the re-organization of therapy space if these are the issues hindering practice change. In the context of inpatient stroke rehabilitation, employing multiple facilitators recruited from core health professional groups involved in interdisciplinary stroke rehabilitation teams was considered a novel and potentially effective strategy for enabling guideline implementation.

In rehabilitation research, guideline provision combined with interactive educational sessions to review best practices has been associated with improved performance of recommended practices compared to mailing of the guideline among rehabilitation providers [24, 25]. Previous trials [24–26], however, have targeted adherence to ≤10 recommendations in one professional group and provided limited description of clustering effects and adjustment for covariates. No studies examined use of local facilitators from different professional groups to promote guideline implementation. The Stroke Canada Optimization of Rehabilitation by Evidence Implementation Trial (SCORE-IT) was a cluster randomized trial designed to evaluate the extent to which a multi-modal, facilitated KT approach to implementing a stroke rehabilitation guideline was more likely than passive strategies, such as mailing the guideline and educational materials, to improve patient function in the inpatient rehabilitation hospital setting (Bayley MT, Wood-Dauphinee S, Richards CL, Salbach NM, Desrosiers J, Eng JJ, et al.: Facilitated knowledge translation improves stroke rehabilitation outcomes: The SCORE-IT cluster randomized controlled trial, under review) [27]. Among patients with stroke treated at facilitated KT sites (*n* = 410), the odds of demonstrating a high level of functional walking capacity, measured using the 6-min walk test, were 1.63 times (95% CI: 1.23–2.17) the odds observed among patients at passive KT sites (*n* = 367) (Bayley MT, Wood-Dauphinee S, Richards CL, Salbach NM, Desrosiers J, Eng JJ, et al.: Facilitated knowledge translation improves stroke rehabilitation outcomes: The SCORE-IT cluster randomized controlled trial, under review). The facilitated KT intervention was not associated with gross manual dexterity (measured using the Box and Block Test) among patients with stroke (OR: 1.69, 95% CI: 0.72–4.01). A mixed methods process evaluation was completed to help explain the results related to patient outcomes. In the qualitative process evaluation [27], focus groups were conducted with 33 nurses, therapists, and managers from 11 of the 20 study sites in the facilitated and passive KT groups to explore their experiences with SCORE-IT. The qualitative analysis yielded four themes describing factors that facilitated or hindered implementation of the KT interventions and clinical integration of the recommended treatments [27]. Themes included: presence/absence of facilitation, agreement that the intervention was practical, familiarity with the recommended treatments, and environmental factors (e.g., staff turnover, lack of space or equipment) [27]. Facilitating factors, such as the presence of an individual who provided stroke teams with support and motivation throughout the trial, and experience with using some of the treatment interventions, were described by participants in both study groups [27]. A fifth theme, namely improved team communication and interdisciplinary collaboration, was identified as an unexpected positive trial outcome that served to facilitate the clinical application of treatment interventions in both study groups [27]. While results from the qualitative process evaluation have increased our understanding of site, provider and treatment characteristics that may have influenced implementation of the study interventions and recommended treatments, the extent to which each recommended treatment was implemented has not been reported and may help to explain why the facilitated KT intervention was associated with improved functional walking capacity but not gross manual dexterity among patients. Thus, this paper presents the results of a quantitative process evaluation of SCORE-IT designed to evaluate the extent to which stroke teams implemented the recommended treatments targeting upper extremity (UE) and lower extremity (LE) motor function, postural control, and mobility in each intervention group.

## Methods

A national, 2-parallel group cluster-randomized, pragmatic trial was conducted from 2007 to 2009. The effectiveness of a facilitated and passive KT intervention for implementing a stroke rehabilitation guideline was evaluated by comparing patient outcomes related to walking capacity and manual dexterity post-intervention. To understand how implementation of guideline recommendations may have influenced study outcomes related to patient function, stroke teams in each intervention group were asked to complete self-report checklists to record their implementation of 18 recommended treatments with each patient seen over a 2-week period pre- and post-intervention. The ethics board at each site and affiliated university approved the study protocol.

### Eligibility and recruitment

Sites with designated rehabilitation beds, an interdisciplinary stroke team with at least one nurse, one physical therapist (PT) and one occupational therapist (OT), and regular inpatients post-stroke (i.e., ≥1 person post-stroke on the unit daily), were considered eligible. Sites with these characteristics were targeted as the treatment recommendations were developed for implementation primarily by nurses, PTs, and OTs in an inpatient rehabilitation setting [13]. Recruitment involved study leads sending letters of invitation to site managers/physiatrists and interviewing to screen for eligibility and obtain consent. All therapists and nurses working on the stroke rehabilitation unit were eligible to participate. A research assistant (RA) hired for each site obtained informed consent from rehabilitation providers. Details of patient eligibility and recruitment are described elsewhere (Bayley MT, Wood-Dauphinee S, Richards CL, Salbach NM, Desrosiers J, Eng JJ, et al.: Facilitated knowledge translation improves stroke rehabilitation outcomes: The SCORE-IT cluster randomized controlled trial, under review).

### Data collection

Following recruitment, site representatives were asked to complete a site readiness checklist that required them to provide information on the language of documentation (English/French), university affiliation (full/partial or none), rehabilitation unit location (freestanding/integrated with a general hospital), and stroke patient volume (expected number of stroke patients/year). Site RAs abstracted patient sociodemographic and clinical data from health records.

The outcome was change in the percentage of patients for which inpatient stroke teams implemented each recommended treatment pre- to post-intervention. All inpatients with stroke were expected to need the majority of treatments. For select treatments that are applied only when indicated (e.g., to reduce hand edema/shoulder pain), a similar proportion in each group was expected to require each treatment owing to randomization. A self-report checklist was created for therapists to document name, profession, and which of the 18 recommended treatments was implemented for each patient. To mitigate social desirability bias [28], a section was added to the checklist where therapists could indicate that a treatment would have been implemented if time had permitted. A similar checklist was created for nurses to report on implementation of 7 treatments (sit-to-stand training, use of LE external support, walking practice, UE range of motion and/or stretching, interventions to prevent shoulder pain, task-specific training of the UE, and education of patients/caregivers on how to handle the affected UE). Site RAs oriented therapists and nurses, who were blinded to study hypotheses, to the checklists and asked them to complete a checklist at the end of every treatment session with patients post-stroke during a two-week period pre- and post-intervention.

### Randomization

A biostatistician, not involved in study recruitment or data collection, used R™ statistical software to stratify hospitals by language of documentation (English/French) and randomly assign them to either the facilitated or passive KT group using a 1:1 allocation ratio. Site staff were informed of their group assignment following completion of pre-intervention data collection on treatment implementation.

### Interventions

The SCORE-IT interventions are described in detail elsewhere (Bayley MT, Wood-Dauphinee S, Richards CL, Salbach NM, Desrosiers J, Eng JJ, et al.: Facilitated knowledge translation improves stroke rehabilitation outcomes: The SCORE-IT cluster randomized controlled trial, under review) [27]. Intervention development was guided by the KTA process and by the results of a qualitative study in which implementation of the stroke rehabilitation guideline was piloted at five inpatient rehabilitation hospitals in Canada [13]. Analysis of transcripts from focus groups involving 79 rehabilitation professionals (physical and occupational therapists, nurses, and directors/managers) identified lack of time, staffing issues, training/education, therapy selection and prioritization, equipment availability, and team functioning/communication as key barriers to guideline implementation. In alignment with the KTA process, the facilitated KT intervention was designed to address these barriers. The facilitated KT intervention included funding for two local facilitators, one nurse and one therapist, to provide 4 h per week of protected time to support guideline implementation over a 16-month period. Having a facilitator from both nursing and allied health was expected to facilitate interdisciplinary collaboration and address barriers related to team functioning and communication [13]. Facilitators attended a two-day workshop where they received media releases for promoting the guideline among clinicians, slide presentations of the treatment protocols, and training in how to apply treatments and run small group education/training sessions. This was designed to prepare facilitators to run local small group education/training sessions to address barriers related to inadequate education/training in how to apply the treatments in clinical practice for existing and new staff. Facilitators were also provided with an outline of strategies used to foster guideline implementation in the pilot study [13], a practice-change toolkit [29], and education in change management. They completed activities to compare current with recommended practice, identify barriers to practice change, and develop a plan that incorporated behavior change strategies to address local challenges to guideline implementation. This was expected to prepare facilitators to address other site-specific barriers related to, for example, insufficient equipment and motivation to change practice [13]. Stroke teams were provided with SCORE guideline booklets that included treatment recommendations and evidence-based treatment protocols and pocket reminder cards designed for therapists or nurses to enable quick and easy access to descriptions of protocols. These resources were expected to address barriers related to inadequate knowledge of and time to read recommendations [13]. Teleconferences and a web-based platform were provided for facilitators to communicate and share successful strategies.

Sites in the passive group received SCORE guideline booklets that did not include treatment protocols, and a handbook [30] and educational video on the use of standardized assessment tools post-stroke. Clinicians were invited to join a list serve to ask questions and share their experiences with the trial.

### Sample size

Post hoc power calculations were performed. Given 1381 observations available to analyze treatment implementation by nurses and therapists, accounting for clustering of observations within patients (mean patient-level intracluster correlation coefficient (ICC) across treatments of 0.12; mean cluster size of 8 observations per patient) yielded an effective sample size of 751 independent observations (375 per group) [31]. With 375 observations per group (2-sided alpha = 0.05) and a baseline implementation rate of 30%, there was 80% power to detect a between-group difference of 10% in the rate of treatment implementation.

Given 547 observations available to analyze treatment implementation by therapists alone, accounting for clustering of observations within patients (mean patient-level ICC across treatments of 0.09; mean cluster size of four observations per patient) yielded an effective sample size of 431 independent observations (215 per group). With 215 observations per group (2-sided alpha = 0.05) and a baseline implementation rate of 10%, there was 80% power to detect a between-group difference of 10% in the rate of treatment implementation.

### Analysis

The unit of analysis was a binary variable that represented whether a patient received a recommended treatment or not during a treatment session. To account for potential clustering effects at the level of the hospital, provider, and patient, a random-effects logistic regression analysis was carried out in SAS v9.3. The analysis included the following steps. First, estimates of the unadjusted rate of treatment implementation within each group pre- and post-intervention, change pre- to post-intervention, and between-group comparison of change were obtained using proc. nlmixed. Next, tests for random variation at the site, provider and patient levels were performed. A significant test result (*α* = 0.05) in more than 33% of models was the criterion for including a clustering variable in all final models.

A final model was constructed for each treatment with intervention group, evaluation time (pre or post), an interaction term of group by time, clustering variables and covariates entered as independent variables using proc. glimmix. Covariates included site location (freestanding/integrated with a general hospital), and size of stroke service (expected #stroke patients/year), and patient motor function (Functional Independence Measure [32] (FIM) motor subscore) and comorbidity (Charlson score [33]) on admission. A significant interaction term (*α* = 0.05) was used to indicate whether change in the extent to which patients received a treatment was greater following the facilitated than following the passive KT intervention after adjusting for clustering and covariates.

## Results

**Fig. 1:**
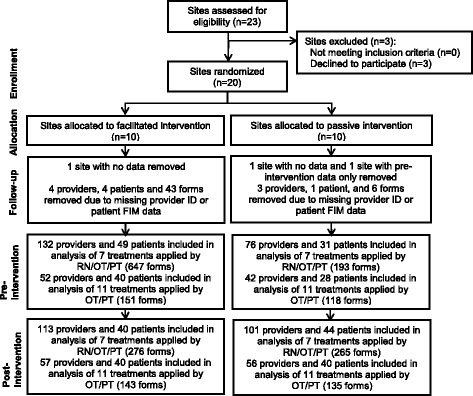
CONSORT flowchart

Figure 1 presents the CONSORT diagram describing the results of recruitment, randomization, and data collection. Of the 20 participating sites, 10 were randomized to the facilitated KT intervention and 10 were randomized to the passive KT intervention. Facilitators from all sites in the facilitated group attended the training workshop (Bayley MT, Wood-Dauphinee S, Richards CL, Salbach NM, Desrosiers J, Eng JJ, et al.: Facilitated knowledge translation improves stroke rehabilitation outcomes: The SCORE-IT cluster randomized controlled trial, under review). Three sites were removed from the analysis because they had no data (*n* = 2) or only pre-intervention data (*n* = 1) due to technical issues with the database. Of the three sites removed, two were from the passive group and were non-academic, and located in a general hospital with 86 and 35 expected patients with stroke/year. The third site removed was from the facilitated group; it was a freestanding site, partially-affiliated with a university, with an expected volume of 90 patients with stroke/year. Thus, data from nine and eight sites in the facilitated and passive group, respectively, were analyzed. The CONSORT diagram indicates the number of unique providers and patients involved in this process evaluation, and the number of checklists submitted by providers pre- and post-intervention by study group.

Table 1 describes characteristics of sites that provided process data. Just over half of the sites in the facilitated and passive groups had no academic affiliation and were freestanding rehabilitation hospitals. The expected number of patients with stroke admitted per year for sites in the facilitated and passive groups was, on average, 95 and 105, respectively. Table 2 describes characteristics of the patients for whom treatment implementation checklists were completed by intervention group and sampling time point. The median age of patients ranged from 62 to 73 years (depending on group, timepoint, and received treatments), with the majority being men (52–69%), ischemic stroke (64–76%), with some arm (CMSA median 2–3) and leg impairment (CMSA median 3). Charlson comorbidity score and the proportion of patients with ischemic stroke were significantly higher in the passive group pre-intervention and post-intervention, respectively.

**Table 1:** Site characteristics

Characteristic	Intervention group	
	Facilitated (*n* = 9)	Passive (*n* = 8)
English-language, *n* (%)	7 (78)	7 (88)
Academic affiliation, *n* (%)		
None	5 (56)	4 (50)
Partial	1 (11)	1 (13)
Full	3 (33)	3 (38)
Freestanding, *n* (%)	5 (56)	4 (50)
Expected number of stroke patients/year, mean ± SD (Range)	95 ± 49 (22–160)	105 ± 72 (30–210)

**Table 2:** Patient characteristics on site admission by intervention group and sampling time point

Characteristic	7 Treatments Implemented by RNs, OTs, and PTs				11 Treatments implemented by OTs, and PTs			
(score range/units)	Facilitated group		Passive group		Facilitated group		Passive group	
	Pre^*^	Post^*^	Pre^*^	Post^*^	Pre^*^	Post^*^	Pre^*^	Post^*^
Patients, *n*	49	40	31	44	40	40	28	40
Age in years	62 (57–77)	68 (60–78)	71 (62–79)	72 (65–79)	64 (57–77)	68 (60–78)	73 (62–79)	72 (64–79)
Men, *n* (%)	34 (69)	26 (65)	16 (52)	25 (57)	27 (68)	26 (65)	15 (54)	22 (55)
Type of stroke,^†^ *n* (%)								
Ischemic	37 (76)	25 (64)^‡^	20 (67)	30 (68)^‡^	30 (75)	25 (64)^‡^	19 (70)	26 (65)^‡^
Hemorrhagic	8 (16)	12 (31)	6 (20)	5 (11)	6 (15)	12 (31)	6 (22)	5 (13)
Unspecified	4 (8)	2 (5)	4 (13)	9 (20)	4 (10)	2 (5)	2 (7)	9 (23)
Side of stroke, *n* (%)								
Right	25 (51)	25 (63)	14 (45)	20 (45)	20 (50)	25 (63)	13 (46)	18 (45)
Left	22 (45)	14 (35)	15 (48)	23 (52)	19 (48)	14 (35)	13 (46)	21 (53)
Brainstem	2 (4)	1 (3)	2 (6)	1 (2)	1 (3)	1 (3)	2 (7)	1 (3)
Days post-stroke on admission	20 (13–28)	15 (9–26)	23 (11–41)	16 (10–34)	20 (13–28)	15 (9–26)	24 (11–44)	15 (10–29)
Charlson Index (0–33)	2 (1–3)^§^	2 (1–3)	3 (2–4)^§^	3 (2–4)	2 (1–3)^‡^	2 (1–3)	3 (2–4)^‡^	3 (1–4)
CMSA Arm^||^ (1–7)	2 (1–3)	3 (2–5)	2 (2–4)	2 (2–5)	2 (1–4)	3 (2–5)	2 (2–4)	2 (2–5)
CMSA Leg^||^ (1–7)	3 (3–4)	3 (3–5)	3 (2–4)	3 (3–4)	3 (3–5)	3 (3–5)	3 (3–4)	3 (3–4)
FIM motor (1–91)	33 (24–54)	46 (36–59)	35 (24–57)	48 (35–59)	32 (22–56)	46 (36–59)	41 (25–58)	49 (35–61)

*Abbreviations*: *CMSA* Chedoke McMaster Stroke Assessment [30], *FIM* functional independence measure [32]

^*^Values are median (P_25_-P_75_) unless otherwise specified

^†^Data from 1 to 2 patients/analysis missing

^‡^Between-group difference, *p* < 0.050

^§^Between-group difference, *p* < 0.010

^||^Data from 13 to 17 patients/analysis missing

Additional file 1: Table S1 describes checklist completion by provider group (see Additional file 1). Nurses contributed the greatest percentage of checklists in the facilitated KT group (50 and 42%, pre- and post-intervention, respectively) and in the passive KT group (39 and 49%, pre- and post-intervention, respectively) for treatments that RNs, OTs, and PTs were asked to apply. Additional file 1: Table S2 provides the ICC value for the effect of clustering on treatment implementation at the site, provider and patient level for each of the 18 treatments. A significant effect of clustering on treatment implementation was observed at the site, provider and patient level in 0, 67, and 39% of models, respectively. The median ICC across treatments for sites, providers and patients was 0.06, 0.21, and 0.08, respectively.

Additional file 1: Table S3 presents mean cluster sizes in terms of the number of providers per site, number of patients per provider, and number of checklist forms per patient pre- and post-intervention by study group (see Additional file 1). Cluster sizes at the site level indicated that the average number of providers contributing data to the analysis across groups and timepoints ranged from 10 to 15 for 7 treatments implemented by RNs, OTs, and PTs and from 5 to 7 for 11 treatments implemented by OTs and PTs. The average number of patients per provider contributing data to the analysis across groups, timepoints, and treatments ranged from 2 to 3. The average number of checklists completed per patient in the analysis across groups and timepoints ranged from 6 to 13 for 7 treatments implemented by RNs, OTs, and PTs and from 3 to 4 for 11 treatments implemented by OTs and PTs.

### Outcomes

Table 3 presents unadjusted estimates of the percentage of patients receiving each treatment pre- and post-intervention, the change in the percentage, and the between-group comparison. Seven of the 18 treatments, including training of sit-to-stand, sitting balance, and standing balance, task-specific training (i.e., stairs), walking practice, interventions to prevent shoulder pain, and task-specific training (i.e., self-care tasks), were being implemented at least 15% of the time in both groups at baseline.

**Table 3:** Unadjusted intervention effect on change in implementation of 18 treatments

Treatment	Time	Estimated % of times implemented (95% CI)				Effect
		*n*	Facilitated (F) group	*n*	Passive (P) group	(Change_F_-Change_P_) % (95% CI)
1. Sit-to-stand^*†‡§^	Pre	647	20.4 (17.3, 23.5)	193	36.3 (29.5, 43.1)	
	Post	276	39.1 (33.4, 44.9)	265	33.6 (27.9, 39.3)	
	Change		*18.7 (12.2, 25.3)*		−2.7 (−11.5, 6.2)	*21.4 (10.4, 32.4)*
2. LE ROM and/or stretching (i.e. to prevent spasticity and contractures)^‡^	Pre	151	15.9 (10.1, 21.7)	118	8.5 (3.4, 13.5)	
	Post	143	10.5 (5.5, 15.5)	135	17.8 (11.3, 24.2)	
	Change		−5.4 (−13.1, 2.3)		*9.3 (1.1, 17.5)*	*−14.7 (−26.0, −3.5)* ^*||*^
3. Use of LE external support (i.e. brace)^‡§^	Pre	647	7.3 (5.3, 9.3)	193	15.0 (10.0, 20.1)	
	Post	276	8.7 (5.4, 12.0)	265	17.4 (12.8, 21.9)	
	Change		1.4 (−2.5, 5.3)		2.3 (−4.5, 9.1)	−0.9 (−8.7, 6.9)
4. Task-specific training (i.e. stairs)^*†‡^	Pre	151	31.8 (24.3, 39.2)	118	26.3 (18.3, 34.2)	
	Post	143	38.5 (30.5, 46.5)	135	37.8 (29.6, 46.0)	
	Change		6.7 (−4.3, 17.6)		*11.5 (0.1, 22.9)*	−4.8 (−20.6, 11.0)
5. Training for sitting balance^*†^	Pre	151	23.8 (17.0, 30.7)	118	17.0 (10.2, 23.7)	
	Post	143	17.5 (11.2, 23.7)	135	25.2 (17.9, 32.5)	
	Change		−6.4 (−15.6, 2.9)		8.2 (−1.8, 18.2)	*−14.6 (−28.2, −1.0)* ^*||*^
6. Training for standing balance^†‡^	Pre	151	51.7 (43.7, 59.6)	118	36.4 (27.7, 45.1)	
	Post	143	52.5 (44.2, 60.7)	135	60.0 (51.7, 68.3)	
	Change		0.8 (−10.7, 12.2)		*23.6 (11.6, 35.6)*	*−22.8 (−39.4, −6.2)*
7. FES for the LE^†^	Pre	151	0.7 (−0.6, 2.0)	118	0 (0, 0)	
	Post	143	0.7 (−0.7, 2.1)	135	0.7 (−0.7, 2.2)	
	Change		0 (−1.9, 1.9)		0.7 (−0.7, 2.2)	−0.7 (−3.1, 1.7)
8. Walking practice^†‡§^	Pre	647	15.9 (13.1, 18.7)	193	31.6 (25.0, 38.2)	
	Post	276	39.1 (33.4, 44.9)	265	32.8 (27.2, 38.5)	
	Change		*23.2 (16.8, 29.6)*		1.2 (−7.4, 9.9)	*22.0 (11.2, 32.8)*
9. Treadmill walking practice^†^	Pre	151	2.7 (0.1, 5.2)	118	6.8 (2.2, 11.3)	
	Post	143	1.4 (−0.5, 3.3)	135	5.2 (1.4, 8.9)	
	Change		−1.3 (−4.5, 2.0)		−1.6 (−7.5, 4.3)	0.3 (−6.4 7.1)
10. UE ROM and/or stretching (i.e. to prevent spasticity and contractures)^*†‡§^	Pre	647	12.7 (10.1, 15.2)	193	21.8 (15.9, 27.6)	
	Post	276	21.4 (16.5, 26.2)	265	25.3 (20.1, 30.5)	
	Change		*8.7 (3.2, 14.2)*		3.5 (−4.3, 11.4)	5.2 (−4.4, 14.7)
11. Interventions to prevent shoulder pain (i.e. sling)^*‡§^	Pre	647	25.0 (21.7, 28.4)	193	25.4 (19.2, 31.5)	
	Post	276	25.7 (20.6, 30.9)	265	21.1 (16.2, 26.1)	
	Change		0.7 (−5.5, 6.8)		−4.3 (−12.1, 3.6)	4.9 (−5.1, 14.9)
12. Task-specific training (i.e. self-care tasks)^*†‡§^	Pre	647	28.9 (25.4, 32.4)	193	37.3 (30.5, 44.1)	
	Post	276	40.9 (35.1, 46.8)	265	43.4 (37.4, 49.4)	
	Change		*12.0 (5.3, 18.8)*		6.1 (−3.0, 15.2)	6.0 (−5.4, 17.3)
13. Techniques to reduce hand edema^*†§^	Pre	151	7.3 (3.1, 11.4)	118	10.2 (4.7, 15.6)	
	Post	143	5.6 (1.8, 9.4)	135	8.9 (4.1, 13.7)	
	Change		−1.7 (−7.3, 3.9)		−1.3 (−8.6, 6.0)	0 (−9.6, 8.8)
14. Ice/heat or soft tissue massage for shoulder	Pre	151	1.3 (−0.5, 3.2)	118	8.5 (3.4, 13.5)	
	Post	143	2.8 (0.1, 5.5)	135	5.2 (1.4, 8.9)	
	Change		1.5 (−1.8, 4.7)		−3.3 (−9.6, 3.0)	4.8 (−2.3, 11.8)
15. FES for wrist/arm/shoulder^*†^	Pre	151	2.0 (−0.2, 4.2)	118	2.5 (−0.3, 5.4)	
	Post	143	1.4 (−0.5, 3.3)	135	1.5 (−0.6, 3.5)	
	Change		−0.6 (−3.5, 2.4)		−1.1 (−4.6, 2.4)	0.5 (−4.1, 5.1)
16. Educate patient or caregiver on how to handle arm or shoulder^*†‡^	Pre	647	8.8 (6.6, 11.0)	193	13.0 (8.2, 17.7)	
	Post	276	9.4 (6.0, 12.9)	265	10.2 (6.5, 13.8)	
	Change		0.6 (−3.5, 4.7)		−2.8 (−8.8, 3.2)	3.4 (−3.9, 10.6)
17. UE constraint-induced therapy	Pre	151	4.6 (1.3, 8.0)	118	10.2 (4.7, 15.6)	
	Post	143	0.7 (−0.7, 2.1)	135	4.4 (1.0, 7.9)	
	Change		*−3.9 (−7.6, −0.3)*		−5.7 (−12.2, 0.8)	1.8 (−5.6, 9.2)
18. Visual imagery to enhance arm recovery^‡^	Pre	151	2.7 (0.1, 5.2)	118	5.1 (1.1, 9.1)	
	Post	143	6.3 (2.3, 10.3)	135	5.2 (1.4, 8.9)	
	Change		3.6 (−1.1, 8.4)		0.1 (−5.4, 5.6)	3.5 (−3.7, 10.8)

Abbreviations: *n* number of observations, *CI* confidence interval, *LE* lower extremity, *ROM* range of motion, *FES* functional electrical stimulation, *UE* upper extremity

Italic text indicates statistically significant results

^*^Demonstration and opportunity to practice during change management workshop

^†^Clinical protocol provided in SCORE guideline

^‡^Clustering effect at provider level

^§^Clustering effect at patient level

^||^No longer significant after adjusting for clustering at the provider and patient level

After adjusting for clustering at patient and provider levels and covariates, the facilitated KT intervention was associated with a significant improvement in the implementation of sit-to-stand training (*p* = 0.028) and walking practice (*p* = 0.043), and the passive KT intervention was associated with improved implementation of standing balance training (*p* = 0.037). Adjustment for the stratification variable did not change the interpretation of the results. Further analysis of which provider groups changed their practice (see Additional file 1: Table S4) revealed that the unadjusted percentage of patients receiving sit-to-stand training was higher post- compared to pre-intervention for nurses (30 vs 10%), and PTs (67 vs 49%) in the facilitated group, and for PTs (58 vs 39%) in the passive group. The unadjusted percentage of patients receiving walking practice was higher post- compared to pre-intervention for nurses (14 vs 6%), OTs (37 vs 21%) and PTs (80 vs 70%) in the facilitated group, and for nurses (14 vs 11%), OTs (25 vs 14%) and PTs (76 vs 68%) in the passive group. The unadjusted percentage of patients receiving standing balance training was higher post- compared to pre-intervention for OTs (36 vs 34%) in the facilitated group, and OTs (38 vs 27%), and PTs (82 vs 45%) in the passive group.

## Discussion

This is among the first process evaluations of a guideline implementation trial involving the use of interprofessional local facilitators in rehabilitation. Findings indicate that a facilitated KT intervention, with local nurse and therapist facilitators, tailoring of strategies to address local barriers, and a guideline with treatment protocols, was of limited effectiveness compared to passive guideline dissemination in improving short-term uptake of a comprehensive guideline by inpatient stroke rehabilitation teams. The process evaluation revealed that the facilitated KT intervention was associated with improved application of only two treatments (sit-to-stand training, walking practice), whereas the passive KT intervention was associated with improved application of one treatment (standing balance training). Results from this process evaluation suggest that superior functional walking capacity observed among patients post-stroke following the facilitated compared to the passive KT intervention (Bayley MT, Wood-Dauphinee S, Richards CL, Salbach NM, Desrosiers J, Eng JJ, et al.: Facilitated knowledge translation improves stroke rehabilitation outcomes: The SCORE-IT cluster randomized controlled trial, under review) resulted from an improved application of sit-to-stand and walking training by stroke teams. Process evaluation findings also indicate that the facilitated KT intervention was not associated with improved gross manual dexterity among patients because this intervention was not associated with improved uptake of treatments targeting UE function.

Results from the current evaluation combined with findings from the qualitative process evaluation of SCORE-IT [27] may help to explain why sit-to-stand and walking practice were more likely than other treatments to be adopted in the facilitated KT group. Facilitation, specifically support and motivation that individuals provided to staff at sites in each group, was perceived to promote the implementation of the recommended treatments [27]. It is possible that facilitation of sit-to-stand and walking training was provided more consistently across sites in the facilitated KT group than in the passive KT group. Results of the qualitative analysis also showed that both familiarity and agreement with recommended treatments because they are “clear and practical to follow” [34, 35] likely helped to promote their uptake [27]. Sit-to-stand and walking training were implemented in at least 15% of patients in each group at baseline (unadjusted estimates) which suggests that some providers had the expertise to perform these treatments and considered them relevant. Sit-to-stand and walking training are also simple, task-oriented mobility treatments that are relevant to daily living. Complex treatments that either involve multiple steps (UE constraint-induced therapy) or technology (functional electrical stimulation, treadmill training) were rarely implemented at baseline and demonstrated either no change or reduced application post-intervention despite being supported by Level A evidence (i.e., evidence from at least one randomized controlled trial, meta-analysis, or systematic review). Based on the SCORE-IT qualitative findings, this was likely because health professionals found that these treatments were time-consuming, and required special training or equipment that was not consistently available across sites [27]. Finally, asking nurses to apply sit-to-stand and walking training, in addition to therapists, appeared an effective facilitated KT strategy as the percentage of patients receiving sit-to-stand and walking practice by nurses increased by 20% (vs a decrease of 11% in the control group) and 8% (vs 3% in the control group), respectively. Improved team communication and interprofessional collaboration were noted as an unintended outcome of SCORE-IT [27]. The improved practice of nurses, likely supported by the nurse facilitator in the facilitated KT group, was particularly influential in the current study as nurses provided a large proportion of the treatment data in the multivariable analysis.

Standing balance training, which increased in the passive KT group, is also a simple task-oriented treatment. Because providers receiving facilitated KT were implementing standing balance training at a high rate at baseline (68% for PTs), they may have prioritized improving adoption of other treatments [13]. Results from the qualitative sub-study [27] indicate that a greater degree of facilitation of and/or agreement with the practicality of standing balance training in the passive compared to the facilitated group, may help to explain why this practice improved in the context of passive dissemination of the stroke rehabilitation guideline.

Despite multiple strategies and resources, the facilitated KT intervention was unsuccessful in improving integration of 18 treatments concurrently. The facilitated approach may not have adequately addressed barriers to integrating numerous treatments simultaneously and complex treatments that were unfamiliar to providers. Targeting fewer treatments and providing individual hands-on training and access to an expert may be a more effective approach based on results from previous guideline implementation trials for low back pain [24] and whiplash [26] rehabilitation.

### Limitations

The primary limitation of this process evaluation was the use of self-report measures of practice that cannot capture clinical judgment or patient preferences and are vulnerable to over-reporting. This limitation, however, would have affected both groups similarly. It could not be determined if treatments received were appropriate due to the unavailability of clinical data at the time implementation was evaluated. Results provide average rates of implementation after controlling for patient and hospital characteristics to optimize comparability between groups.

## Conclusions

A facilitated KT intervention incorporating a guideline with treatment protocols and trained local nurse and therapist facilitators was of limited effectiveness compared to passive guideline dissemination in improving short-term uptake of a comprehensive guideline by inpatient stroke rehabilitation teams. Conducting this process evaluation as part of the trial was valuable as it revealed the nature of the practice change, in terms of the type of health providers involved and the type of and extent to which treatments were implemented, underpinning patient function outcomes observed in the main analysis. The combination of quantitative and qualitative process evaluation findings provided a basis for hypothesis generation related to designing KT interventions to overcome challenges to integrating treatments recommended in stroke rehabilitation guidelines in the context of interdisciplinary team care. Specifically, KT strategies that better address the need for staff training and team functioning to account for treatment complexity and prioritization post-stroke may be needed. Finally, the study design and analytical approach described in the current study, which involved consideration of multi-level clustering effects, and adjustment for site- and patient-level covariates, is innovative and will help to advance the field of implementation science in the context of rehabilitation guideline implementation.

## Additional file

Additional file 1: Table S1. Checklist completion by provider group. **Table S2.** Intracluster correlation coefficients for the 18 recommended treatments. **Table S3.** Cluster sizes at the site, provider and patient level by study group and sampling time point. **Table S4.** Unadjusted rate of treatment implementation by healthcare professional and group pre- and post-intervention. (DOCX 21 kb)

## Abbreviations

CI: Confidence Interval
CMSA: Chedoke McMaster Stroke Assessment
CONSORT: Consolidated Standards of Reporting Trials
FES: Functional Electrical Stimulation
FIM: Functional Independence Measure
ICC: Intracluster Correlation Coefficient
KT: Knowledge Translation
KTA: Knowledge to Action
LE: Lower Extremity
OR: Odds Ratio
OT: Occupational Therapist
PARiHS: Promoting Action on Research Implementation
PT: Physical Therapist
RA: Research Assistant
RN: Registered Nurse
ROM: Range of Motion
SCORE-IT: Stroke Canada Optimization of Rehabilitation by Evidence-Implementation Trial
UE: Upper Extremity
