# Supplementary material for: Facilitated interprofessional implementation of a physical rehabilitation guideline for stroke in inpatient settings: process evaluation of a cluster randomized trial
Source: Implement Sci. 2017 Aug 1;12:100. doi: 10.1186/s13012-017-0631-7 (PMC5539742; doi:10.1186/s13012-017-0631-7)
Supplement: Additional file 1: Table S1. — Checklist completion by provider group. Table S2. Intracluster correlation coefficients for the 18 recommended treatments. Table S3. Cluster sizes at the site, provider and patient level by study group and sampling time point. Table S4. Unadjusted rate of treatment implementation by healthcare professional and group pre- and post-intervention. (DOCX 21 kb) [file 13012_2017_631_MOESM1_ESM.docx]

Additional file 1 Table S1 Checklist completion by provider group

| **Characteristic** | **Intervention Group** | |
| --- | --- | --- |
|  | **Facilitated** | **Passive** |
| Checklist completion by provider group pre/post-intervention for treatments completed by RNs, OTs, PTs,^*^ n (%) |  |  |
| RN | 326 (50)/116 (42) | 75 (39)/129 (49) |
| OT | 77 (12)/71 (26) | 49 (25)/56 (21) |
| PT | 73 (11)/76 (28) | 62 (32)/67 (25) |
| Other^†^ | 171 (26)/13 (5) | 7 (4)/13 (5) |
| Checklist completion by provider group pre/post-intervention for treatments completed by OTs, PTs,^‡^ n (%) |  |  |
| OT | 76 (50)/69 (48) | 49 (42)/56 (41) |
| PT | 73 (48)/73 (51) | 62 (53)/67 (50) |
| Other^†^ | 2 (1)/1 (1) | 7 (6)/12 (9) |

Abbreviations: RN, nurse; OT, occupational therapist; PT, physical therapist.

^*^Significant difference between groups pre-intervention, chi-squared value=98.8, p<0.0001.

^†^E.g., recreation therapists, speech-language pathologists.

^‡^Significant difference between groups post-intervention, Fisher’s exact test, p=0.003.

Table S2 Intracluster correlation coefficients for the 18 recommended treatments

| **Treatment** | **Intracluster Correlation Coefficient** | | |
| --- | --- | --- | --- |
|  | **Site** | **Provider** | **Patient** |
| 1. Sit-to-stand | 0.06 | **0.26** | 0.10 |
| 2. LE ROM and/or stretching | 0.00 | **0.30** | 0.04 |
| 3. Use of LE external support | 0.11 | 0.10 | **0.23** |
| 4. Task-specific training (i.e., stairs) | 0.00 | **0.35** | 0.00 |
| 5. Training for sitting balance | 0.01 | 0.16 | 0.09 |
| 6. Training for standing balance | 0.02 | **0.23** | 0.08 |
| 7. FES for the LE | 0.12 | 0.00 | 0.00 |
| 8. Walking practice | 0.02 | **0.31** | 0.11 |
| 9. Treadmill walking practice | 0.03 | 0.19 | 0.17 |
| 10. UE ROM and/or stretching | 0.08 | **0.32** | 0.06 |
| 11. Interventions to prevent shoulder pain | 0.08 | 0.16 | **0.21** |
| 12. Task-specific training (i.e., self-care tasks) | 0.05 | **0.28** | 0.06 |
| 13. Techniques to reduce hand edema | 0.06 | 0.06 | **0.22** |
| 14. Ice/heat or soft tissue massage for shoulder | 0.14 | 0.16 | 0.20 |
| 15. FES for wrist/ arm/shoulder | **0.24** | 0.16 | 0.04 |
| 16. Educate patient or caregiver on how to handle arm or shoulder | 0.05 | **0.25** | 0.07 |
| 17. UE constraint-induced therapy | 0.11 | 0.19 | 0.14 |
| 18. Visual imagery to enhance arm recovery | 0.00 | **0.31** | 0.04 |

Abbreviations: LE, lower extremity; ROM, range of motion; FES, functional electrical stimulation; UE, upper extremity.

Note: ICC values >0.20 are bolded.

Table 3 Cluster sizes at the site, provider and patient level by study group and sampling time point

| **Cluster** | **Cluster Size Mean (SD)** | | | | | | | |
| --- | --- | --- | --- | --- | --- | --- | --- | --- |
| **Level** | **7 Treatments Implemented by RNs, OTs, and PTs** | | | | **11 Treatments Implemented by OTs and PTs** | | | |
|  | **Facilitated** | | **Passive** | | **Facilitated** | | **Passive** | |
|  | **Pre** | **Post** | **Pre** | **Post** | **Pre** | **Post** | **Pre** | **Post** |
| Site (no. providers/site) | 15 (8) | 13 (6) | 10 (7) | 13 (7) | 6 (3) | 6 (3) | 5 (4) | 7 (4) |
| Provider (no. patients/provider) | 3 (3) | 2 (1) | 2 (1) | 2 (1) | 2 (1) | 2 (1) | 2 (1) | 2 (1) |
| Patient (no. forms/patient) | 13 (13) | 7 (3) | 6 (4) | 6 (3) | 4 (2) | 4 (1) | 4 (2) | 3 (1) |

Abbreviations: SD, standard deviation; RN, nurse; OT, occupational therapist; PT, physical therapist; no., number.

Table S4. Percentage of times treatments were implemented by provider and intervention group pre- and post-intervention, unadjusted for clustering

| **Treatment** | **Provider** | **Facilitated Intervention** | | | | **Passive Intervention** | | | |
| --- | --- | --- | --- | --- | --- | --- | --- | --- | --- |
|  |  | **Pre** | | **Post** | | **Pre** | | **Post** | |
|  |  | **#forms** | **% of forms indicating treatment implemented** | **#forms** | **% of forms indicating treatment implemented** | **#forms** | **% of forms indicating treatment implemented** | **#forms** | **% of forms indicating treatment implemented** |
| 1. Sit-to-stand | RN | 326 | 10 | 116 | 30 | 75 | 32 | 129 | 21 |
|  | OT | 77 | 39 | 71 | 25 | 49 | 41 | 56 | 32 |
|  | PT | 73 | 49 | 76 | 67 | 62 | 39 | 67 | 58 |
|  | Other | 171 | 20 | 13 | 31 | 7 | 29 | 13 | 38 |
| 2. LE ROM and/or stretching | OT | 76 | 11 | 69 | 1 | 49 | 2 | 56 | 0 |
|  | PT | 73 | 21 | 73 | 19 | 62 | 15 | 67 | 28 |
|  | Other | 2 | 50 | 1 | 0 | 7 | 0 | 12 | 42 |
| 3. Use of LE external support | RN | 326 | 4 | 116 | 7 | 75 | 17 | 129 | 13 |
|  | OT | 77 | 6 | 71 | 7 | 49 | 0 | 56 | 9 |
|  | PT | 73 | 10 | 76 | 11 | 62 | 26 | 67 | 34 |
|  | Other | 171 | 12 | 13 | 23 | 7 | 0 | 13 | 8 |
| 4. Task-specific training (i.e. stairs) | OT | 76 | 13 | 69 | 12 | 49 | 8 | 56 | 18 |
|  | PT | 73 | 52 | 73 | 63 | 62 | 44 | 67 | 55 |
|  | Other | 2 | 0 | 1 | 100 | 7 | 0 | 12 | 33 |
| 5. Training for sitting balance | OT | 76 | 12 | 69 | 12 | 49 | 29 | 56 | 18 |
|  | PT | 73 | 36 | 73 | 23 | 62 | 10 | 67 | 30 |
|  | Other | 2 | 50 | 1 | 0 | 7 | 0 | 12 | 33 |
| 6. Training for standing balance | OT | 76 | 34 | 69 | 36 | 49 | 27 | 56 | 38 |
|  | PT | 73 | 68 | 73 | 68 | 62 | 45 | 67 | 82 |
|  | Other | 2 | 100 | 1 | 0 | 7 | 29 | 12 | 42 |
| 7. FES for the LE | OT | 76 | 0 | 69 | 0 | 49 | 0 | 56 | 0 |
|  | PT | 73 | 1 | 73 | 1 | 62 | 0 | 67 | 1 |
|  | Other | 2 | 0 | 1 | 0 | 7 | 0 | 12 | 0 |
| 8. Walking practice | RN | 326 | 6 | 116 | 14 | 75 | 11 | 129 | 14 |
|  | OT | 77 | 21 | 71 | 37 | 49 | 14 | 56 | 25 |
|  | PT | 73 | 70 | 76 | 80 | 62 | 68 | 67 | 76 |
|  | Other | 171 | 11 | 13 | 38 | 7 | 57 | 13 | 31 |
| 9. Treadmill walking practice | OT | 76 | 1 | 69 | 0 | 49 | 0 | 56 | 0 |
|  | PT | 73 | 4 | 73 | 3 | 62 | 13 | 67 | 9 |
|  | Other | 2 | 0 | 1 | 0 | 7 | 0 | 12 | 8 |
| 10. UE ROM and/or stretching | RN | 326 | 3 | 116 | 3 | 75 | 9 | 129 | 8 |
|  | OT | 77 | 49 | 71 | 51 | 49 | 45 | 56 | 64 |
|  | PT | 73 | 25 | 76 | 22 | 62 | 21 | 67 | 27 |
|  | Other | 171 | 10 | 13 | 15 | 7 | 0 | 13 | 23 |
| 11. Interventions to prevent shoulder pain | RN | 326 | 21 | 116 | 30 | 75 | 36 | 129 | 33 |
|  | OT | 77 | 18 | 71 | 21 | 49 | 18 | 56 | 11 |
|  | PT | 73 | 36 | 76 | 24 | 62 | 21 | 67 | 9 |
|  | Other | 171 | 32 | 13 | 23 | 7 | 0 | 13 | 8 |
| 12. Task-specific training (i.e. self-care tasks) | RN | 326 | 28 | 116 | 53 | 75 | 60 | 129 | 66 |
|  | OT | 77 | 40 | 71 | 45 | 49 | 41 | 56 | 36 |
|  | PT | 73 | 14 | 76 | 12 | 62 | 10 | 67 | 15 |
|  | Other | 171 | 32 | 13 | 77 | 7 | 14 | 13 | 0 |
| 13. Techniques to reduce hand edema | OT | 76 | 9 | 69 | 10 | 49 | 20 | 56 | 16 |
|  | PT | 73 | 5 | 73 | 1 | 62 | 3 | 67 | 4 |
|  | Other | 2 | 0 | 1 | 0 | 7 | 0 | 12 | 0 |
| 14. Ice/heat or soft tissue massage for shoulder | OT | 76 | 3 | 69 | 6 | 49 | 4 | 56 | 13 |
|  | PT | 73 | 0 | 73 | 0 | 62 | 13 | 67 | 0 |
|  | Other | 2 | 0 | 1 | 0 | 7 | 0 | 12 | 0 |
| 15. FES for wrist/ arm/shoulder | OT | 76 | 4 | 69 | 3 | 49 | 4 | 56 | 2 |
|  | PT | 73 | 0 | 73 | 0 | 62 | 2 | 67 | 1 |
|  | Other | 2 | 0 | 1 | 0 | 7 | 0 | 12 | 0 |
| 16. Educate patient or caregiver on how to handle arm or shoulder | RN | 326 | 4 | 116 | 3 | 75 | 7 | 129 | 8 |
|  | OT | 77 | 26 | 71 | 17 | 49 | 31 | 56 | 21 |
|  | PT | 73 | 27 | 76 | 13 | 62 | 8 | 67 | 7 |
|  | Other | 171 | 3 | 13 | 0 | 7 | 0 | 13 | 0 |
| 17. UE constraint-induced therapy | OT | 76 | 7 | 69 | 1 | 49 | 22 | 56 | 5 |
|  | PT | 73 | 3 | 73 | 0 | 62 | 0 | 67 | 4 |
|  | Other | 2 | 0 | 1 | 0 | 7 | 14 | 12 | 0 |
| 18. Visual imagery to enhance arm recovery | OT | 76 | 4 | 69 | 12 | 49 | 12 | 56 | 11 |
|  | PT | 73 | 1 | 73 | 1 | 62 | 0 | 67 | 1 |
|  | Other | 2 | 0 | 1 | 0 | 7 | 0 | 12 | 0 |

Abbreviations: RN, nurse; OT, occupational therapist; PT, physical therapist; LE, lower extremity; ROM, range of motion; FES, functional electrical stimulation; UE, upper extremity.
